# Profiling microRNAs through development of the parasitic nematode *Haemonchus* identifies nematode-specific miRNAs that suppress larval development

**DOI:** 10.1038/s41598-019-54154-6

**Published:** 2019-11-26

**Authors:** Neil D. Marks, Alan D. Winter, Henry Y. Gu, Kirsty Maitland, Victoria Gillan, Martin Ambroz, Axel Martinelli, Roz Laing, Rachel MacLellan, Jessica Towne, Brett Roberts, Eve Hanks, Eileen Devaney, Collette Britton

**Affiliations:** 1 Institute of Biodiversity, Animal Health and Comparative Medicine, College of Medical, Veterinary and Life Sciences, University of Glasgow, Bearsden Road, Glasgow, G61 1QH UK; 2 Department of Biochemical Sciences, Faculty of Pharmacy, Charles University, Hradec Kralove, Czech Republic; 3 Wellcome Sanger Institute, Wellcome Genome Campus, Hinxton, Cambridgeshire CB10 1SA UK; 4 Present Address: West of Scotland Genetic Services, Level 2B, Laboratory Medicine, Queen Elizabeth University Hospital, Govan Road, Glasgow, G51 4TF UK; 5 Present Address: Global Station for Zoonosis Control, Global Institution for Collaborative Research and Education (GI-CoRE), Hokkaido University, N20 W10 Kita-ku, Sapporo Japan; 6 Present Address: Wellcome Centre for Integrative Parasitology, Institute of Infection, Immunity and Inflammation, College of Medical, Veterinary and Life Sciences, University Avenue, Glasgow, G12 8QQ UK

**Keywords:** Parasite development, miRNAs

## Abstract

Parasitic nematodes transition between dramatically different free-living and parasitic stages, with correctly timed development and migration crucial to successful completion of their lifecycle. However little is known of the mechanisms controlling these transitions. microRNAs (miRNAs) negatively regulate gene expression post-transcriptionally and regulate development of diverse organisms. Here we used microarrays to determine the expression profile of miRNAs through development and in gut tissue of the pathogenic nematode *Haemonchus contortus*. Two miRNAs, *mir*-*228* and *mir*-*235*, were enriched in infective L3 larvae, an arrested stage analogous to *Caenorhabditis elegans* dauer larvae. We hypothesized that these miRNAs may suppress development and maintain arrest. Consistent with this, inhibitors of these miRNAs promoted *H*. *contortus* development from L3 to L4 stage, while genetic deletion of *C*. *elegans* homologous miRNAs reduced dauer arrest. Epistasis studies with *C*. *elegans daf*-*2* mutants showed that *mir*-*228* and *mir*-*235* synergise with FOXO transcription factor DAF-16 in the insulin signaling pathway. Target prediction suggests that these miRNAs suppress metabolic and transcription factor activity required for development. Our results provide novel insight into the expression and functions of specific miRNAs in regulating nematode development and identify miRNAs and their target genes as potential therapeutic targets to limit parasite survival within the host.

## Introduction

For parasitic nematodes, identifying the mechanisms regulating co-ordinated expression of mRNA transcripts is important in understanding parasite development in response to varying host or environmental conditions. Here we focus on changes in microRNA (miRNA) levels during development of the pathogenic gastrointestinal nematode *Haemonchus contortus*. miRNAs are small (21–23 nucleotides) non-coding RNAs found in animals, plants and viruses that regulate gene expression post-transcriptionally. miRNAs bind with partial sequence complementarity to sites, most often in the 3′ untranslated region (UTR) of their target mRNAs^1^, resulting in translational repression and mRNA degradation^2^. The association of specific miRNAs with physiological and pathological conditions has led to their exploitation as biomarkers of disease^3^ and development of miRNA mimics and inhibitors as novel therapeutics^4,5^.

miRNAs were first identified in the free-living nematode *Caenorhabditis elegans*, with *lin*-*4* and *let*-*7* shown to be essential regulators of development^6,7^. Individual loss of most *C*. *elegans* miRNAs leads to no obvious phenotype under laboratory conditions^8^. Neverthless, miRNAs have subtle roles in specific processes or under stress conditions, as identified using specific assays^9–12^. While there is a wealth of data on *C*. *elegans* miRNA expression and function, little is known of miRNA function in parasitic nematodes. The availability of genome sequence data for a number of helminth species has enabled identification of parasite miRNAs by deep sequencing and computational approaches (reviewed in^13^). We previously identified 192 miRNAs in *H*. *contortus* by deep sequencing of small RNAs expressed in infective L3 and mixed sex adult worms^14^. Identifying in more detail when and where these are expressed, and the genes they target, will help reveal the regulatory mechanisms controlling parasite development and adaptation to the host environment.

For many parasitic nematodes, infection of vertebrate hosts is initiated by the infective L3 stage, considered analogous to the developmentally arrested dauer larvae of *C*. *elegans* that form in response to unfavourable conditions (starvation and crowding)^15^. In *C*. *elegans*, development is promoted by the insulin/IGF-1 and TGF-β signaling pathways, that ultimately lead to binding of steroid hormones to the nuclear hormone receptor DAF-12^16^. While DAF-12 and components of the insulin signaling pathway, *daf*-*2* and *daf*-*16*, are conserved in parasitic nematodes^17–19^, the mechanisms that regulate parasite larval activation and development have yet to be identified^20^.

The aim of this work was to profile the expression of miRNAs through different developmental stages of a gastrointestinal parasitic nematode, and determine the potential targets and functions of some of these. We focused on *H*. *contortus*, a highly pathogenic, blood-feeding parasite of small ruminants with a global distribution. Genome and transcriptome information is well-advanced for *H*. *contortus*^21,22^, with the genome assembled in chromosomes^23^, making it an important model for related nematodes, including human hookworms^24^. As with other parasitic nematodes, control of *H*. *contortus* infection relies largely on treatment with broad-spectrum anthelmintic drugs, but the efficacy of these is under serious threat from drug resistance^25^. Identifying the regulatory molecules and pathways essential for nematode development has the potential to lead to novel therapeutics for nematode control.

## Results

### Microarray profiling of *H*. *contortus* miRNAs

**Figure 1:**
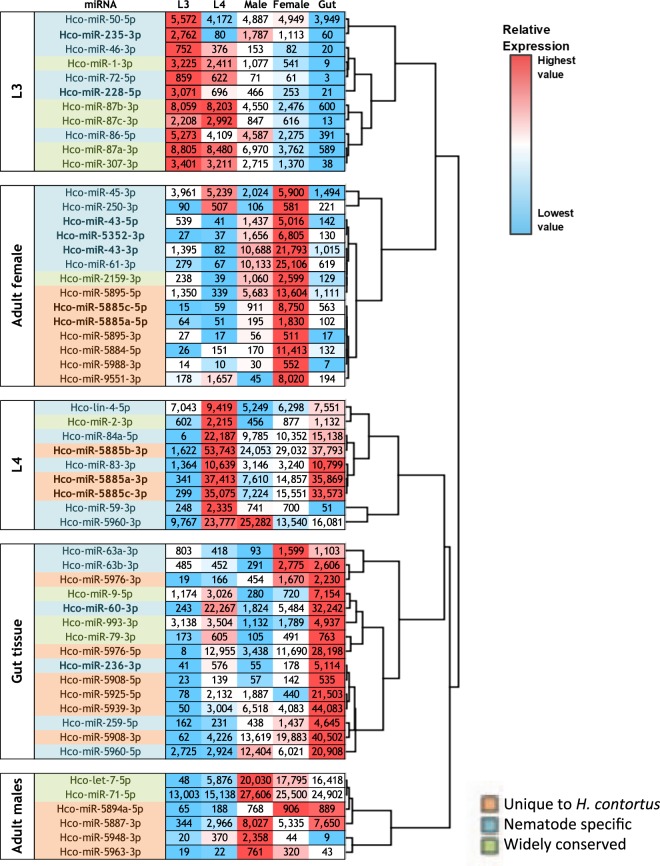
Differential expression of *H*. *contortus* miRNAs from microarray data. Rooted dendogram of complete linkage hierarchical clustering of miRNAs showing differential expression between at least one developmental stage or in adult female gut tissue, identified by ANOVA. Corrected normalised data with microarray threshold signal of >500 fluorescent units and p-value < 0.05. Groups are labeled according to life-cycle stage or gut tissue showing the highest level of expression. Data are displayed using a heat-map and miRNA names are color-coded according to conservation status, as indicated. Note that *hco*-*mir*-*5885a/b/c*-*3p*, enriched in L4 stage, is homologous to *cel*-*mir*-*58* and *Drosophila* bantam.

A custom microarray containing probes to *H*. *contortus* and *C*. *elegans* miRNAs (Methods) was screened with RNA from five *H*. *contortus* life-cycle stages: sheathed infective L3, exsheathed L3 (exsheathed in 5% sodium hypochlorite solution and cultured *in vitro* at 37 °C for 24 h), L4 larvae collected from the abomasum of infected sheep 7 days post-infection, and adult male and egg-producing female worms collected 28 days post-infection. In addition, the array was probed with RNA from gut tissue dissected from adult female worms. Patterns of expression for *hco*-*let*-*7* and *hco*-*lin*-*4* were similar to that reported in *C*. *elegans*^26^ supporting the reliability of the data (Fig. 1 and Supplementary Table S1). From normalised data, ANOVA identified 55 miRNAs showing significant variation in expression (p < 0.05; microarray fluorescent signal >500) between at least two life-cycle stages or between adult whole worm extract and gut tissue. No difference was found for any probe between sheathed versus exsheathed L3, consistent with the reported paucity of changes in *H*. *contortus* mRNA expression following L3 exsheathment by the same method^21^; only data for sheathed L3 were examined further. Hierarchical clustering (Fig. 1) identified five major miRNA groups based upon their enriched expression in each life-cycle stage or in adult female gut tissue. Based on our criteria, most differentially expressed miRNAs were conserved in other nematodes (47%) or in different phyla (30%), with only 23% unique to *H*. *contortus* (miRBase release 21) (Supplementary Fig. 1). Of the lowest expressed miRNAs on the array (fluorescent signal <500), most (91%) have been found to date only in *H*. *contortus*. These miRNAs may not yet have developed significant functional roles or, alternatively, may be expressed in specific cells or tissues, and therefore of low abundance.

### *H*. *contortus* miRNAs are developmentally and spatially regulated

**Figure 2:**
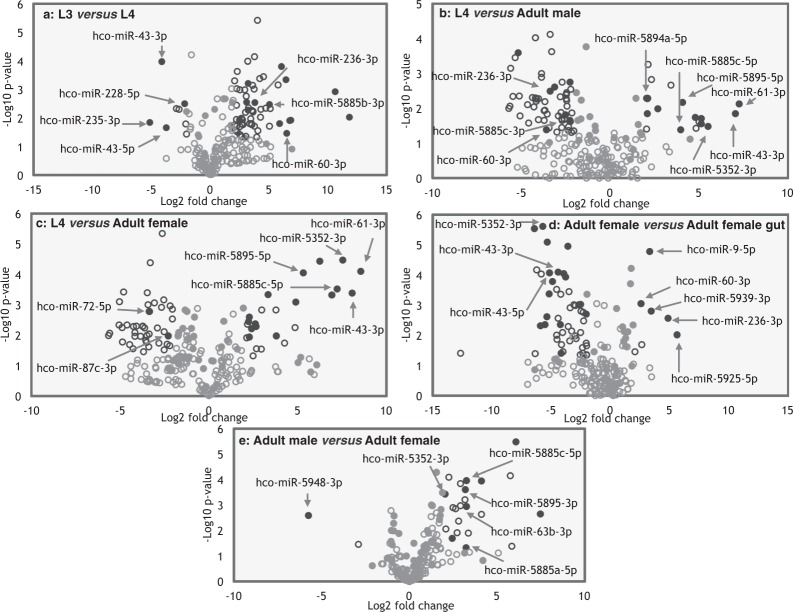
*H*. *contortus* miRNAs are temporally and spatially regulated. Volcano plots of pairwise comparison of microarray data between sequential life-cycle stages, male and female adults and between adult female extract and adult female gut tissue. (**a**–**e**) Black filled circles indicate miRNAs with pairwise t-test P-values < 0.05 and Log_2_ fold change >2, grey filled circles indicate miRNAs not significantly differentially expressed between stages, black open circles represent probes significantly differentially expressed but with signal <500 and grey open circles indicate probes with signal <500 and not differentially expressed. A selection of miRNAs are labeled, including those referred to in Results.

**Figure 3:**
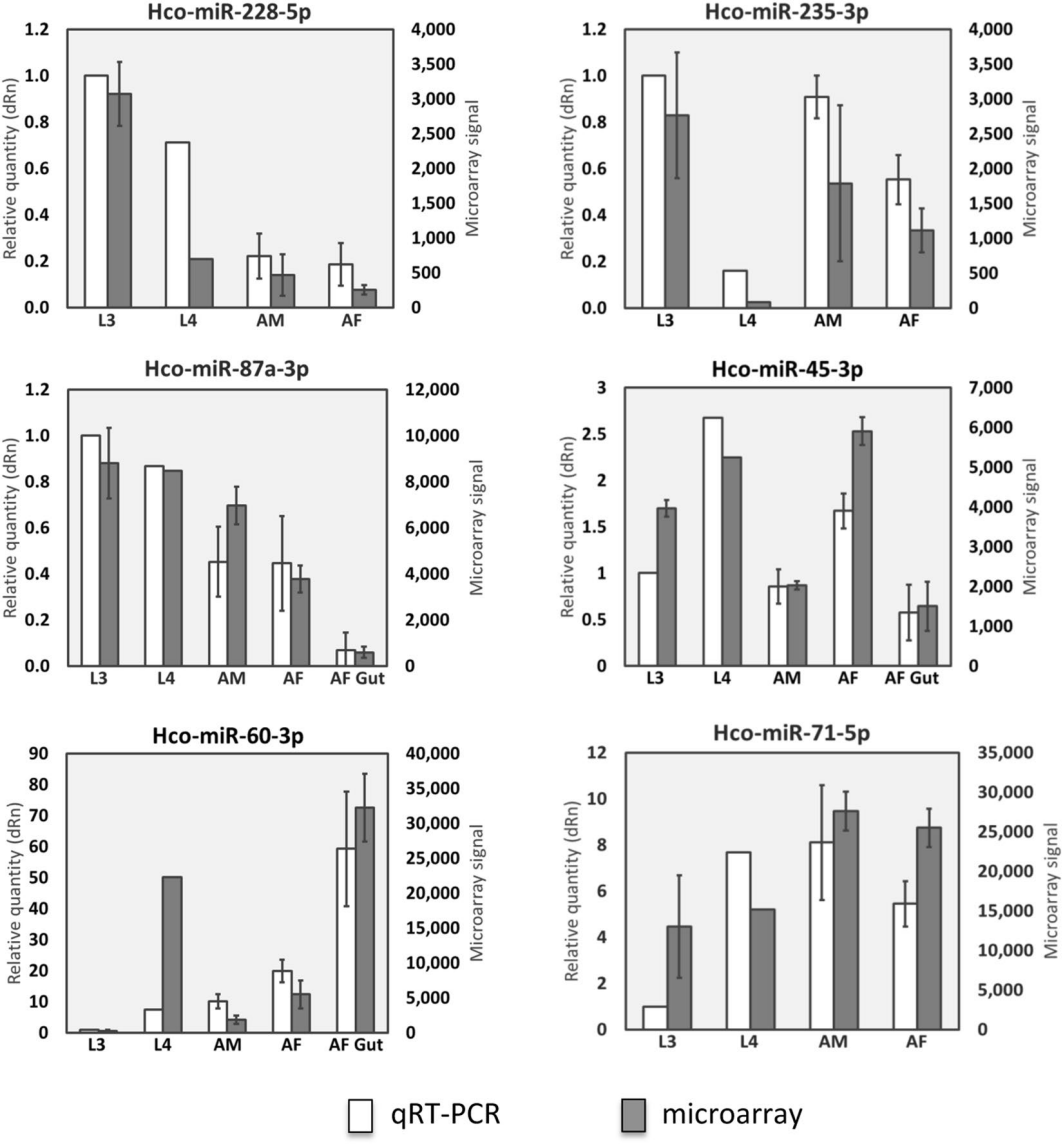
qPCR confirmation of microarray data. qPCR data (white bars) are shown as relative quantities (dRn), where the calibrator stage (L3) was set to one in all graphs. Microarray data (grey bars) are shown as signal strength. Data from three biological replicates are shown, except for L4 stage where duplicate samples were used. Error bars represent ± standard deviation. Data for gut tissue are shown for *hco*-*miR*-*87a*-*3p*, *hco*-*miR*-*45*-*3p* and *hco*-*miR*-*60*-*3p* only.

Pairwise t-test comparisons (p-values < 0.05; Log_2_ fold change >2) identified four miRNAs with significantly greater abundance in infective L3 relative to L4 stage (volcano plot Fig. 2a and Supplementary Table S3). Of these, expression of *hco*-*mir*-*228*-*5p* and *hco*-*mir*-*235*-*3p* peaked in L3 (Fig. 1), while most *H*. *contortus* miRNAs were not abundantly expressed in L3 larvae. Fourteen miRNAs were significantly more abundant in L4 relative to L3 stage (Fig. 2a and Supplementary Table S3), while 11 and 16 miRNAs were significantly upregulated in adult male or female worms, respectively, relative to L4 (Fig. 2b,c, Supplementary Table S3), suggesting possible roles in reproduction or in adult worm survival/lifespan. Five miRNAs were more abundant in adult female gut tissue relative to adult female whole worm extract (see Fig. 2d and Supplementary Table S3), two of which were enriched in L4 stage, suggesting possible roles in gut development (*hco*-*mir*-*60*-*3p and hco*-*mir*-*236*-*3p*). Blood-feeding begins in L4 larvae and development from L3 to L4 is marked by enlargement of the gut lumen and a switch in metabolic pathways^21^. In *C*. *elegans*, *mir*-*60 and mir*-*236* are also gut expressed^27^, suggesting similar regulatory functions across nematode species. Only a single miRNA, *hco*-*mir*-*5948*-*3p*, was enriched in adult males relative to adult female worms (Fig. 2e). qPCR of selected miRNAs showed good correlation with the microarray data (Fig. 3). For *hco*-*mir*-*228*-*5p* and *hco*-*mir*-*235*-*3p* (peak in L3), both were expressed in the pre-infective L1 stage, but at a lower level than in L3 (57% and 49% of L3 level, respectively) (Supplementary Fig. 2).

Some of the miRNAs profiled here by microarray were also identified in a recent study by Ma *et al*.^28^ using small RNA library sequencing to characterise the miRNAome of L3, exsheathed L3 and *in vitro* generated L4 of *H*. *contortus*. Fewer miRNAs were found (88) but for those identified in both studies, the developmental expression patterns were similar. In the present study, in addition to confirming upregulation of *hco*-*mir*-*5976*-*5p* in L4 versus L3 stage^28^, we also show that this miRNA is abundant in gut tissue (Fig. 1).

### Effects of mimics and inhibitors on *H*. *contortus* L3 to L4 development

**Figure 4:**
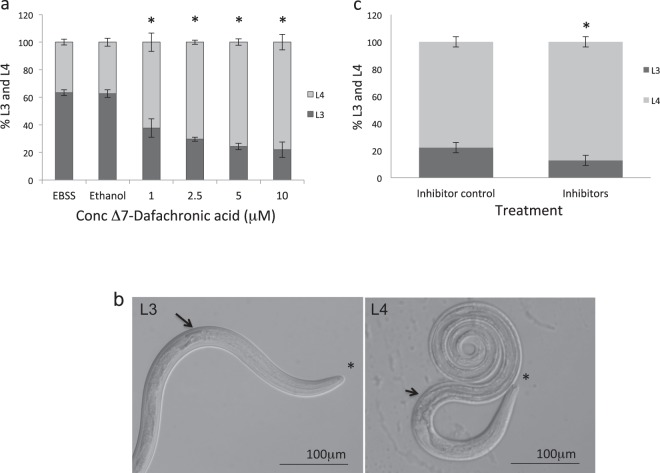
Activation and inhibition of *H*. *contortus* L3 to L4 development *in vitro*. (**a**) Δ7-Dafachronic acid (Δ7-DA) was added to EBSS culture medium/5% CO_2_/37 °C at 1–10 μM final concentration and development from L3 to L4 stage examined microscopically. Control wells contained the same volume of ethanol (DA solvent). Percentage of *H*. *contortus* L3 and L4 larvae after 72 h in the presence or absence of DA is shown. Significant differences were observed at all DA concentrations (two tailed t-test P = 0.0039 to P = 0.000056). Mean and standard deviation of three experiments are shown. (**b**) Morphology of L3 and L4 stages *in vitro*. Dark gut granules (arrow) and closed buccal cavity (asterisk) are observed in L3 larvae, while L4 stage show an open gut lumen (arrowhead) and open buccal cavity (asterisk). (**c**) Percentage of *H*. *contortus* L3 and L4 larvae after five days in the presence of inhibitors of both *hco*-*mir*-*228* and *hco*-*mir*-*235* (final concentration 2 μM). Inhibitors enhanced development from L3 to L4 relative to control inhibitor at same final concentration (t-test *P = 0.038). Mean and standard deviation of three replicate experiments are shown.

We hypothesised that the elevated expression of *mir*-*228* and *mir*-*235* in L3 may be required to maintain arrest prior to host infection. Effects of interfering with or mimicking *hco*-*mir*-*228* and *hco*-*mir*-*235* activity on *H*. *contortus* larval development *in vitro* were examined. In an effort to enhance *H*. *contortus* L3 to L4 development *in vitro*, we tested effects of the DAF-12 ligand dafachronic acid (DA) on larval development^29^. Addition of Δ7-DA (final concentration of 1–10 μM) to *H*. *contortus* L3 larvae *in vitro* significantly increased the number of exsheathed L3 that molted to L4, after 48–72 h, relative to EBSS medium/37 °C/5% CO_2_ alone (Fig. 4a; P = 0.0039 at 1 μM DA). L4 larvae were identified by their open buccal cavity and enlarged gut lumen (Fig. 4b). This is similar to recent findings from Ma *et al*.^30^ who achieved L3 to L4 development with exogenous DA and also identified endogenous Δ7-DA in *H*. *contortus* L3 larvae. Consistent with these observations, we identified expression of a homologue of *C*. *elegans daf*-*12* in *H*. *contortus* L3 by RT-PCR (GenBank Accession number MN017114).

Addition of mimics of *hco*-*mir*-*228* and *hco*-*mir*-*235* in combination, either in the presence or absence of DA, did not significantly reduce the rate or number of L3 developing to L4 *in vitro*, relative to cultures treated with a control miRNA mimic (up to 2 μM) (not shown). Conversely, in the presence of inhibitors for both *hco*-*mir*-*228* and *hco*-*mir*-*235* there was a slight, statistically significant (P = 0.038) increase in the percentage of L3 developing to L4 after five days in culture, but not with control inhibitor (concentration 2 μM) (Fig. 4c; 87% L4 with inhibitors; 77% L4 with control inhibitor). Albeit slight, this is the first report, as far as we are aware, of a phenotypic effect of miRNA inhibition in a parasitic nematode and suggests that *hco*-*mir*-*228* and *hco*-*mir*-*235* have an inhibitory effect on larval development that can be observed *in vitro*.

### *mir*-*228* and *mir*-*235* regulate development in *C*. *elegans*

Stable gene knockout is not currently feasible in *H*. *contortus* or most other parasitic nematodes. We adopted a comparative approach and examined effects of genetic deletion of *mir*-*228* and *mir*-*235* on dauer formation in the related clade V nematode *C*. *elegans*. No defects in dauer arrest or development have been reported for these miRNAs, however *cel*-*mir*-*235* is required for maintaining L1 arrest, when eggs hatch in the absence of food^12^, while dietary restriction of *C*. *elegans* adult worms induces expression of *mir*-*235*^31^, *mir*-*228* and *mir*-*71*^32^.

**Figure 5:**
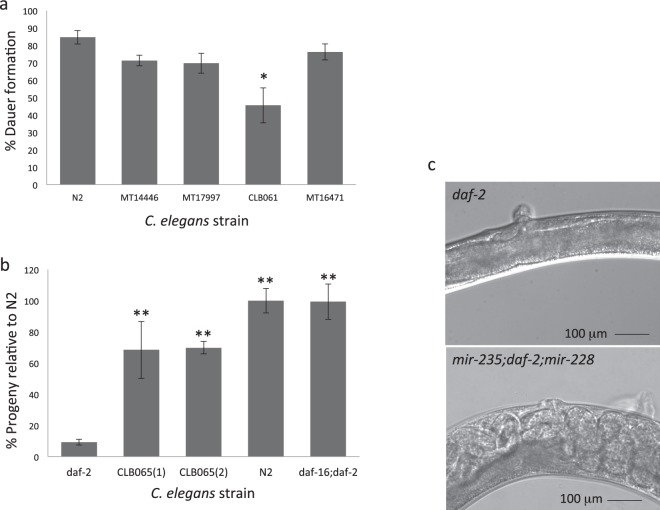
*mir*-*228* and *mir*-*235* regulate *C*. *elegans* dauer arrest and reproductive development. (**a**) Reduction in number of dauers formed in *C*. *elegans* miRNA mutant strains. Dauer formation was assessed in the presence of 2% dauer pheromone at 27 °C for 72 h and calculated as the percentage of larvae resistant to 1% SDS (see Methods). Mean ± standard deviation of three replicate plates is shown. Asterisk indicates significant reduction in dauer formation relative to N2 wild type strain (two-tailed t-test *P = 0.012). Mutant strains tested: MT14446 (*mir*-*228*(*n4382*)*IV*); MT17997(*mir*-*235*(*n4504*)*I*); CBL061(*mir*-*235*(*tn4504*)*I*);(*mir*-*228*(*n4382*)*IV*); MT16471(*mir*-*60*(*n4947*)*II*). (**b**) Suppression of sterility in *C*. *elegans* strain CLB065(*mir*-*235*(*n4504*)*I*; *daf*-*2*(*e1370*)*III*; *mir*-*228*(*n4382*)*IV*. Mean number of progeny produced per adult, 48 h after start of egg laying expressed as % N2 number progeny for *daf*-*2*(*e*-*1370*), triple mutant CLB065 (results for two independent lines 1 and 2) and *daf*-*16*; *daf*-*2 mutant*. Mean and standard deviation of three replicate studies are shown. Asterisks indicate significant increase in number of progeny relative to *daf*-*2*(*e1370*) (two-tailed t-test **P < 0.01). (**c**) Suppression of protruding vulva phenotype in CLB065 triple mutant (*mir*-*235*; *daf*-*2*; *mir*-*228*) compared to *daf*-*2* at x40 magnification. Adult worm shown is representative of approximately 50 adult worms, from three individual experiments.

Genetic deletion of *cel*-*mir*-*228* (strain MT14446) or *cel*-*mir*-*235* (strain MT17997) individually caused an insignificant reduction in dauer formation relative to *C*. *elegans* wild-type N2 strain (Fig. 5a). We generated a *cel*-*mir*-*228* and *cel*-*mir*-*235* double mutant strain (CLB061) and this showed a significant decrease in dauer arrest (46% reduction, P = 0.012), indicating that these miRNAs function together to regulate development. In contrast, deletion of the gut-expressed *cel*-*mir*-*60* (strain MT16471) had no effect on number of dauers (Fig. 5a). Increasing the concentration of pheromone to 3% did not significantly increase the number of dauers formed in the mutant strains, and the double mutant (*cel*-*mir*-*235*; *cel*-*mir*-*228*) showed normal dye filling with the lipophilic dye Dil, indicating normal amphid structure (data not shown).

### Loss of *mir*-*228* and *mir*-*235* can suppress *daf*-*2* loss of function effect

*C*. *elegans* development to reproductive stage requires activation of the DAF-2 insulin receptor and *daf*-*2 loss of function* (*lf*) mutants are dauer constitutive (*daf*-*c*) at restrictive temperature^33^. *Daf*-*2*(*lf*) effects are dependent on DAF-16 FOXO transcription factor, which regulates many genes involved in developmental arrest, reproduction and longevity^34,35^. We investigated whether *cel*-*mir*-*228* and *cel*-*mir*-*235* may synergise with DAF-16 by determining if these miRNAs were required for *daf*-*2*(*lf*) effects. At 25 °C, *daf*-*2*(*e1370*) forms 100% dauers; additional loss of *mir*-*228* and *mir*-*235* (strain CLB065 (*mir*-*235*(*n4504*)*I*; *daf*-*2*(*e1370*)*III*; *mir*-*228*(*n4382*)*IV*) showed no reduction in the highly penetrant *daf*-*c* phenotype at 25 °C. However, at 22.5 °C, *daf*-*2*(*e1370*) develop beyond dauer stage and become sterile adults or adults producing embryonic lethal progeny^36,37^. At 22.5 °C, triple mutant strain CLB065 produced a significantly greater number of viable progeny relative to the *daf*-*2* mutant strain; CLB065 produced 69% of the number of progeny produced by wild-type N2 or *daf*-*16*:*daf*-*2* mutant, significantly more than *daf*-*2* mutant which produced only 9% of the number of progeny produced by N2 or *daf*-*16*; *daf*-*2* mutant (p = 0.005 and p = 0.000018 for CLB065 lines 1 and 2, respectively) (Fig. 5b). In addition, loss of *mir*-*228* and *mir*-*235* in strain CLB065 suppressed the protruding vulva phenotype observed in *daf*-*2*(*e1370*) adults at 22.5 °C (Fig. 5c). Therefore, *mir*-*228* and *mir*-*235* synergise with DAF-16 activity and may act in parallel or downstream of DAF-16 to suppress development. The differences observed at 25 °C and 22.5 °C suggest that *mir*-*228* and *mir*-*235* function at a threshold level of DAF-16: these miRNAs can enhance the effects of DAF-16 and add robustness to DAF-16 activity at non-saturating levels (22.5 °C) but not at saturating, high levels (25 °C), consistent with a role of miRNAs in fine-tuning gene function.

### Predicted targets of *mir*-*228* and *mir*-*235* regulate nematode metabolism and development

Target prediction was carried out using 3′UTR databases for *C*. *elegans* and *H*. *contortus* to identify possible mechanisms by which *mir*-*228* and *mir*-*235* may regulate development. For *cel*-*mir*-*228*, multiple target sites were predicted by PITA^38^ and TargetScan^39^, 56 of which were identified by both algorithms (Supplementary Table S4). These predictions were refined to a subset of 13 high confidence genes (see Methods) (Table 1) and included genes encoding enzymes that function in metabolic pathways, particularly lipid, amino acid and carbohydrate metabolism. For *C*. *elegans mir*-*235*, again multiple targets were predicted using PITA and TargetScan, 87 of which were common to both algorithms (Supplementary Table S5). Five targets satisfied the high confidence criteria (see Methods) and these encoded genes involved in development and lipid storage/metabolism (Table 1). Interestingly, transcription factor ETS-4 is involved in lipid catabolism^40^ and is also a proposed antagonist of DAF-16 function in *C*. *elegans* longevity^41^. This suggests a potential mechanism for *mir*-*235* to enhance DAF-16 activity, by suppressing ETS-4.

**Table 1:** High confidence predicted target genes of *cel*-*mir*-*228* and *cel*-*mir*-*235* using PITA and TargetScan.

*C*. *elegans* gene	Cosmid ID	Known/proposed function (Wormbase)
***miRNA*:** ***cel*-*mir*-*228***		
*mboa***-***2* sterol O-acyltransferase	H19N07.4	Lipid metabolism
transketolase	Y39E4A.3	Mitochondrial enzyme; branched chain keto dehydrogenase (BCKD) component; lifespan
*syg***-***1* cell adhesion molecule	K02E10.8	Synapse formation
*sek***-***1* map kinase kinase	R03G5.2	Innate immune response
glucosidase	T04A8.7	Development; apoptosis
*inx***-***1* innexin	C16E9.4	Thermotaxis; defecation
*dbt***-***1* transacylase	ZK669.4	Larval and embryonic development; BCKD component
*skpo***-***1* peroxidase	F49E12.1	Morphogenesis; innate immunity
*tim***-***1* timeless	Y75B8A.22	Regulation of developmental timing
sulfotransferase	C18B2.2	unknown
*ckr***-***2* neuropeptide receptor	Y39A3B.5	Digestive enzyme secretion; lipid storage; lifespan
*eps***-***8* cell signaling adaptor	Y57G11C.24	Intestinal morphology
N/A	T26C5.2	unknown
***miRNA***: ***cel***-***mir***-*235*		
*rbc***-***1* (RaBConnectin related)	F54E4.1	Re-association of vacuolar ATPase after glucose starvation
N/A	F33D4.6	Lipid storage; larval development; morphogenesis
*zmp***-***2* zinc metalloprotease	H19M22.3	Molting; larval development; disease resistance
*atf***-***2* cAMP-dependent	K08F8.2 transcription factor	Excretory cell development; negative regulator of autophagy and apoptosis
*ets***-***4* transcription factor	F22A3.1	Lipid catabolism; lifespan

GO analysis of the target gene lists for *cel*-*mir*-*228* showed significant enrichment for KEGG pathways relating to metabolism and development, including valine, leucine and isoleucine degradation (Table 2 and Supplementary Table S6); these are branched chain amino acids (BCAAs) that accumulate in different long-lived mutants of *C*. *elegans*^42^. A more limited range of GO terms were enriched for targets predicted for *cel*-*mir*-*235*, including locomotion and alternative splicing (Tables 2 and S7).

**Table 2:** GO enrichment terms for *cel*-*mir*-*228* and *cel*-*mir*-*235* target genes predicted by TargetScan.

Pathway	Gene count	P-value	Benjamini
***miRNA*:** ***cel*-*mir*-*228***			
Valine, leucine and isoleucine degradation	8	7.70E-07	3.80E-05
Developmental protein	16	7.80E-06	1.00E-03
Glycoprotein	18	1.50E-05	9.90E-04
Metabolic pathways	22	7.80E-05	2.00E-03
Propanoate metabolism	5	1.90E-04	3.20E-03
Zinc	20	2.00E-04	8.70E-03
LIM domain	5	2.00E-04	6.50E-03
Glyoxylate and dicarboxylate metabolism	5	2.10E-04	7.60E-03
***miRNA*:** ***cel*-*mir*-*235***			
Alternative splicing	21	5.70E-06	7.40E-04
Body morphogenesis	21	8.60E-06	2.60E-03
Locomotion	34	9.10E-06	1.40E-03
Nucleus	23	4.20E-04	2.70E-02

Target prediction programs PITA, MiRanda and RNAhybrid^38,43,44^ identified predicted binding sites in *H*. *contortus* 3′UTR regions for *hco*-*mir*-*228* or *hco*-*mir*-*235*, using 3′UTR databases we generated (Methods). For *hco*-*mir*-*228*, >1000 target genes were predicted by each program, with 143 genes predicted by all three programs (Supplementary Table S8). For *hco*-*mir*-*235*, multiple targets were again identified, with 133 common to all three gene prediction lists (Supplementary Table S9). In total, *H*. *contortus* homologs of 12 *C*. *elegans* high confidence target genes could be identified in NCBI database and, of these, five had predicted binding sites for *hco*-*mir*-*228* or *hco*-*mir*-*235* (F33D4.6, *zmp*-*2*, *mboa*-*2*, *sek*-*1*, *eps*-*8*) (Supplementary Table S10).

GO analysis of the *hco*-*mir*-*228* and *hco*-*mir*-*235* full target gene list was carried out with *C*. *elegans* putative homologs of *H*. *contortus* target genes as input, where these could be identified. Among the KEGG terms enriched for *hco*-*mir*-*228* MiRanda-predicted targets were valine, leucine and isoleucine degradation, metabolic pathways, propanoate metabolism and carbon metabolism (Supplementary Table S11), similar to the GO terms enriched for *cel*-*mir*-*228* (Table 2). For *hco*-*mir*-*235*, a number of GO terms were enriched for target genes, including locomotion, nematode larval development, oxidoreductase activity, metalloendopeptidase activity, mTOR signaling and aminoacyl tRNA biosynthesis (Supplementary Table S12). These terms are consistent with reduced BCAA degradation, protein translation and TOR signaling during *C*. *elegans* arrest^42^ and suggest that *mir*-*228* and *mir*-*235* in *C*. *elegans* and *H*. *contortus* regulate some of the same metabolic pathways to suppress reproductive development.

### Validation of miRNA-target mRNA interactions

**Figure 6:**
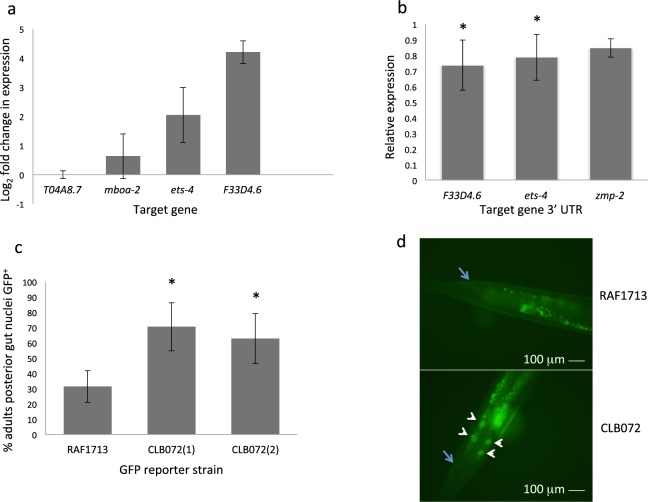
Regulation of target genes by *C*. *elegans mir*-*228* and *mir*-*235*. (**a**) Levels of predicted target genes T04A8.7, H19N07.4 (*mboa*-*2*), F22A3.1 (*ets*-*4*) and F33D4.6 were assessed relative to constitutively expressed *C*. *elegans* actin (*act*-*3*). Data are expressed as Log_2_ fold change in mutant strain CLB061 (*mir*-*235*(*n4504*); *mir*-*228*(*n4382*)) relative to N2 wild-type strain. PCR reactions were carried out in triplicate and the mean of three biological replicate RNA samples from synchronized L4 is shown +/− standard deviation. (**b**) Repression of firefly luciferase in HEK293 cells transfected with the 3′ UTR of predicted targets of *cel*-*mir*-*235* (F33D4.6, *ets*-*4*, *zmp*-*2*). Data are expressed as the mean ratio of three experiments comparing Firefly to Renilla luciferase signal in the presence of *cel*-*mir*-*235* cloned in the forward orientation relative to the reverse orientation, where the reverse is expressed as 1. Error bars indicate standard deviation (two-tailed t-test *P = 0.045 with F33D4.6 3′UTR and P = 0.044 for *ets*-*4* 3′UTR). (**c**) Percentage of adult worms expressing GFP in posterior gut nuclei of strain RAF1713(*rrrSi400* [*Pets*-*4*::*ets*-*4*::*gfp*::*ets*-*4 3*′*UTR*] *II*.; *unc*-*119*(*ed3*) *III*) [71] or strain CLB072 (two independent lines 1 and 2), generated by crossing RAF1713 with MT17997(*mir*-*235*(*n4504*)I. Error bars indicate standard deviation (two-tailed t-test, P = 0.022 and P = 0.047 for CLB072 lines 1 and 2, respectively). (**d**) *ets*-*4*::*GFP* expression in posterior gut nuclei in CLB072 but not in RAF1713, indicating enhanced GFP expression in the absence of *cel*-*mir*-*235*. Images taken at same exposure at x40 magnification. To enable visualisation of GFP expression, RNAi of *ets*-*4* suppressor *rege*-*1* was required^38^ and was carried out by RNAi feeding of both RAF1713 and CLB072 strains, in an identical manner. Arrow indicates rectum, arrowheads indicate GFP^+^ gut nuclei. Smaller autofluorescent gut granules can also been seen.

miRNA:mRNA regulation of targets predicted in *C*. *elegans* was examined using three approaches: qPCR, dual luciferase reporter assay and, for *ets*-*4*, use of a GFP reporter construct. Target gene expression was compared by qPCR using RNA from synchronized *C*. *elegans* L4 stage of CLB061 (*mir*-*235*; *mir*-*228*) and wild-type N2. Expression levels of two predicted targets of *cel*-*mir*-*235* were increased in CLB061 relative to N2 worms (*ets*-*4*; 4 fold (Log_2_ 2.05)) and F33D4.6; 16 fold (Log_2_ 4.2)) (Fig. 6a). There was no detectable increase in expression of two targets of *cel*-*mir*-*228* tested, *mboa*-*2* and T04A8.7, in CLB061 relative to N2 worms.

Interaction between *cel*-*mir*-*228* or *cel*-*mir*-*235* and the 3′UTR of predicted targets was then examined using a dual luciferase reporter assay, as described previously^45^. A significant reduction in reporter expression was observed with the 3′UTRs of two targets tested in the presence of *cel*-*mir*-*235* (F33D4.6, 27% reduction, P = 0.045; *ets*-*4*, 22% reduction, P = 0.044 (Fig. 6b). For the 3′UTRs tested in the presence of *cel*-*mir*-*228*, no detectable reductions in luciferase levels were observed relative to the control (data not shown).

Transcription factor Cel-ETS-4 is a proposed antagonist of DAF-16^41^ and promotes lipid catabolism^40^. To further examine negative regulation of *ets*-*4* by *cel*-*mir*-*235*, expression of a GFP reporter under the control of *ets*-*4* 3′UTR (*C*. *elegans* strain RAF1713)^40^ was compared in the presence and absence of *cel*-*mir*-*235*. This was achieved by genetic crossing of RAF1713 with strain MT17997(*mir*-*235*(*n4504*)) to generate strain CLB072. In both RAF1713 and CLB072, GFP expression was observed in gut cell nuclei, but was observed down the length of the gut only in the *mir*-*235* mutant background (CLB072). In the *mir*-*235* mutant background, GFP expression was stronger and significantly more adult worms showed posterior gut nuclei expression (67% versus 31% in RAF1713; t-test P = 0.022 and p = 0.047 for two independent lines CLB072(1) and CLB072(2)) (Fig. 6c,d). Together, the above data indicate that *cel*-*mir*-*235* can suppress *ets*-*4* via its 3′UTR, and therefore can potentially enhance DAF-16 activity and also repress lipid catabolism associated with development.

## Discussion

For optimal survival, nematodes must respond to environmental cues and co-ordinate developmental programs accordingly, particularly during life-cycle transitions. The importance of epigenetic mechanisms, including DNA methylation^46^, histone acetylation/methylation^47–49^ and post-transcriptional regulation by microRNAs (reviewed in^13^), are beginning to be revealed, but how these modulate parasite development is not known. miRNAs often function together to regulate gene networks to produce a co-ordinated and rapid change in gene expression that ensures robust developmental progression^32^. In this study we profiled the expression of all known miRNAs of *H*. *contortus* and identified those significantly up- or down-regulated during development from infective L3 stage to reproducing adult worms. Pan-nematode or more widely conserved miRNAs were enriched in the L3 and L4 larval stages, suggesting regulation of evolutionary conserved targets. In contrast, almost half of the miRNAs abundantly expressed in adult worms, or in adult gut tissue, are unique to *H*. *contortus* (from miRBase Release 21), which may indicate roles in adaptation and survival within the host. We focused on two miRNAs, *mir*-*228* and *mir*-*235*, significantly enriched in *H*. *contortus* L3 stage that may function to maintain L3 arrest and prevent developmental progression. By generating a *C*. *elegans* mutant of the homologous miRNAs, we showed that they are required together for larval arrest, with formation of significantly fewer dauer larvae. Our data suggest that *mir*-*228* and *mir*-*235* re-inforce activity of FOXO-transcription factor DAF-16 to suppress metabolic pathways required for development.

Sequences with identity or near identity to *mir*-*228* are present in other nematodes, including the filarial parasite *B*. *malayi* and pig roundworm *Ascaris suum* (http://www.mirbase.org) but not in other organisms. *mir*-*235* is restricted to clade V nematodes, however a related miRNA, *mir*-*92*, is expressed in nematode clades III and IV, and is similar to the mammalian *mir*-*17*-*92* cluster. Wang *et al*.^50^ profiled miRNA expression during early development of *A*. *suum* and showed that *mir*-*92* is most abundant in day 21 larvae, at which time larvae are arrested L3 stage. This suggests that *mir*-*235* plays a conserved role in parasitic nematode quiescence prior to host infection. In *C*. *elegans*, *mir*-*235*^12^ and *mir*-*71*^51^ are essential for maintaining L1 arrest, while *mir*-*71* together with *mir*-*34* are required for L1 and dauer arrest, and during adult worm ageing, consistent with roles in stress resistance^52^. *Cel*-*mir*-*235* was also recently shown to be required for longevity of dietary restricted *eat*-*2* mutants by suppressing Wnt signaling, leading to the proposal that *mir*-*235* is a major sensor of different dietary restriction regimens^31^. Our data show that *mir*-*235* is important in suppressing development and maintaining arrest, under starvation or stress conditions, and that this function is conserved in free-living and parasitic nematode species.

Mimics and inhibitors of *mir*-*228* and *mir*-*235* were tested directly on *H*. *contortus* cultured *in vitro*. A significant, albeit slight, increase in L3 to L4 development was observed in the presence of inhibitors, supporting a role for *hco*-*mir*-*228* and *hco*-*mir*-*235* in suppressing development, not only *in vivo* but also during *in vitro* conditions. Consistent with this, Ma *et al*.^28^ observed a decrease in *mir*-*235* transcript level in *in vitro* derived L4 relative to L3 stage. Although small, this is the first report of a phenotypic effect in a parasitic nematode using miRNA inhibitors. The limited effect may reflect inefficient uptake and/or distribution of inhibitor within the worms. It will be important in future studies to determine effects of *mir*-*228* and *mir*-*235* inhibitors and mimics on parasite development and reproduction within the host, as we did previously for RNAi silencing of *H*. *contortus* vaccine antigen aminopeptidase H11^53^.

Effects on ovarian structure of adult liver fluke *Schistosoma japonicum* have been reported following *in vitro* delivery of miRNA inhibitors by electroporation^54^, but we found that this delivery method led to high mortality in *H*. *contortus* larvae. The use of viral vectors to deliver and express parasite genes^55^ and the demonstration of gene knockout in *S*. *stercoralis* using CRISPR/Cas9^56^ should facilitate greater manipulation of parasite miRNA and mRNA levels for more detailed functional analysis. We also demonstrate here that the steroid hormone Δ7-DA can enhance *H*. *contortus* larval development *in vitro*, consistent with recent findings^30^. Improvements to *in vitro* culture conditions, such as this, should help in future studies of small RNA uptake for gene silencing and subsequent phenotypic analysis.

Epistasis studies using mutants of the *C*. *elegans daf*-*2* insulin receptor gene demonstrated interaction between the activities of *mir*-*228*, *mir*-*235* and the insulin signaling pathway. Loss of *mir*-*228* and *mir*-*235* had no effect on the *daf*-*c* phenotype of *daf*-*2*(*e1370*) mutants at the restrictive temperature of 25 °C. However, at semi-permissive temperature (22.5 °C), *daf*-*2*(*e1370*) mutants develop to sterile or near-sterile adults^36,37^ and we show here that this requires *mir*-*228* and *mir*-*235*, as triple mutants develop to reproductive adults. Importantly, the differences observed at 25 °C and 22.5 °C suggest that these miRNAs modulate the effects of transcription factor DAF-16 and reinforce DAF-16 function at a threshold level to ensure robustness of stress response programs. This is consistent with analysis of *daf*-*2* mosaics that show an all or nothing tissue response^37^, suggesting there is a quantitative threshold of signal required to commit to developmental progression or maintain arrest. We propose that *mir*-*228* and *mir*-*235*, and possibly other miRNAs, function with DAF-16 to inhibit progression to the commitment threshold required for development. DAF-2 and DAF-16 are functionally conserved in parasitic nematode species^17–19^. Interestingly, no DAF-2 agonistic ligands have been identified to date in the genome of *H*. *contortus*^57^, suggesting that host signals may drive post-infection development and miRNAs may be important in co-ordinating developmental progression in response to such signals.

Target genes for *mir*-*228* and *mir*-*235* identified computationally were involved in metabolic regulation, in particular lipid metabolism and BCAA degradation. In the *C*. *elegans* dauer stage, lipid stores are abundant initially, while lipid and amino acid metabolism are reduced, while a converse pattern occurs during reproductive growth, characterized by aerobic lipid and carbohydrate metabolism^58^. Transcriptome data for *H*. *contortus* also indicate a developmental switch in metabolic activity, from gluconeogenesis in the L3 stage to oxidative metabolism of carbohydrate and lipid in L4 and adult worms^21^. Lipid metabolism can be modulated by addition of DA, which promotes *H*. *contortus* L3 to L4 development^30^. Analysis of gene clusters differentially expressed during *C*. *elegans* dauer and reproductive development also showed downregulation of genes involved in BCAA and fatty acid degradation, carbon metabolism and glyoxylate and dicarboxylate metabolism after dauer commitment, while genes associated with splicing, ribosome, RNA polymerase were upregulated in L4 stage^59^. The target prediction data presented here suggest that *mir*-*228* and *mir*-*235* are involved in regulating these metabolic switches to suppress development, while, in contrast, DA promotes metabolic and signaling pathways associated with development^30^.

Using different experimental approaches we show that *mir*-*235* suppresses expression of target gene *ets*-*4* and interacts with *ets*-*4* 3′UTR. ETS-4 is a transcription factor that promotes lipid catabolism by increasing expression of lipid metabolic genes^40^. We propose that one function of *mir*-*235* is to suppress *ets*-*4* levels, leading to a reduction in multiple lipid catabolic pathways. In addition, ETS-4 is a putative antagonist of DAF-16^41^ and therefore, by reducing ETS-4, *mir*-*235* may also enhance DAF-16 activity in suppressing development.

In conclusion, we profiled the expression of miRNAs during development of *H*. *contortus* parasitic stages, determined predicted targets of two miRNAs and identified their potential synergism with DAF-16 in regulating nematode metabolism and development. With resistance to current anthelmintics, which predominantly target neurotransmission, becoming an increasing concern, drug repurposing and omics-based approaches are being used to identify small molecules that target metabolic pathway enzymes, as alternative therapeutics^60,61^. Our findings from miRNA expression and target analysis help identify multiple metabolic pathways and switches required for development, some of which may be novel nematode-specific targets for blocking parasite survival and ameliorating disease.

## Methods

### Ethics statement

Animal experiments were carried out at the Moredun Research Institute (MRI), Edinburgh, UK. All procedures were approved by MRI Experiments and Ethics Committee (MRI E46 11) and carried out in accordance with the Animal (Scientific Procedures) Act 1986, UK, UK Home Office PPL 60/03899.

### *H*. *contortus* preparation and maintenance

*H*. *contortus* MHco3(ISE) parasite stages were collected as described^21^. L3 were also exsheathed in 5% sodium hypochlorite solution and cultured in Earle’s Balanced Salt Solution (EBSS, pH 5), containing penicillin (250 U/ml, streptomycin (50 μg/ml), amphotericin B (125 μg/ml) at 37 °C, 5% CO_2_ for 24 h. *H*. *contortus* gut was dissected from 20 adult female worms, as described^62^, washed in PBS then placed in Trizol (Life Technologies).

### Total RNA extraction and miRNA microarray

Parasites were ground in liquid nitrogen and total RNA extracted using Trizol (Invitrogen), with RNA for miRNA microarray prepared with an additional 75% ethanol wash. RNA integrity was assessed using a Bioanalyser 2100 (Agilent). Three biological replicate samples (parasites isolated from different animals) were prepared, except for L4, where two replicates were used. Microarrays were performed by LC Sciences (Texas, USA), using a custom array containing probes to 609 *H*. *contortus* miRNA sequences (all mature and star miRNA sequences we identified previously^14^) and all *C*. *elegans* sequences in miRBase release 16. Microarray data are deposited in NCBI’s Gene Expression Omnibus (GEO Series accession number GSE101501; https://www.ncbi.nlm.nih.gov/geo/query/acc.cgi?acc=GSE101501).

Normalised microarray data was corrected as described^63^, using a microarray threshold signal of >500 fluorescent units and P-value < 0.05. Data was subjected to hierarchical clustering using the matrix visualization and analysis platform GENE-E, version 3.0.240 (http://www.broadinstitute.org/cancer/software/GENE-E/index.html). Row distance was calculated using a one-minus Pearson correlator metric and complete linkage (farthest-neighbour approach). All other settings were default. A rooted dendrogram and three-colour heat-map were generated. Groups were labeled according the life-cycle stage or gut tissue with highest level of expression. Pairwise t-test comparisons were carried using GenePattern software (http://software.broadinstitute.org/cancer/software/genepattern/gene-expression-analysis); t-test P-values of <0.05 and Log_2_ fold changes of >2 were retained. Volcano plots were generated using Multiplot (v2) (http://software.broadinstitute.org/cancer/software/genepattern/modules/docs/Multiplot/2). Conservation of miRNAs was carried out by interrogation of miRBase 21 using the miRNA precursor sequences for all known *H*. *contortus* miRNAs.

### miRNA and mRNA qPCR

miRNA cDNA Synthesis and qPCR Master Mix protocols were followed (Agilent)) using 1 μg DNase I-treated (Ambion) total RNA. RNA samples for L3, adult male and adult female qPCR were independent of those used on microarray. Two independent L1 samples were used in qPCR and compared with L3. Results were normalized to *hco*-*miR*-*5899*-*3p*, a constitutively expressed miRNA. mRNA was prepared from 1 μg of DNase I-treated total RNA, using SuperScript II (Invitrogen) with oligo(dT) primer and qPCR carried out using Brilliant III Ultra-Fast SYBR Green QPCR Master Mix (Agilent) with *C*. *elegans* actin (*act*-*3*) as a normalising gene^64^. qPCR reactions were carried out in triplicate on an Agilent Mx3005P qPCR System. All oligonucleotide sequences are listed in Supplementary Table 13.

### *In vitro* assay of *H*. *contortus* L3 to L4 development

*H*. *contortus* L3 were exsheathed using CO_2_ and incubated in 120 μl Earle’s Balanced Salt Solution (EBSS) containing penicillin (250 U/ml), streptomycin (50 μg/ml) and fungizone (1.25 μg/ml) at 37 °C, 5% CO_2_ in 96 well plates (1.5 L3/ μl). miRIDIAN double-stranded miRNA mimics for *hco*-*mir*-*228* and *hco*-*mir*-*235* were designed by Dharmacon (http://dharmacon.gelifesciences.com) and added together at a final concentration of 2 μM to cultures containing 2.5 μM Δ7-DA. Miridian control mimic #1 (Dharmacon) was added at the same final concentration. miRCURY LNA Power Inhibitors of *hco*-*mir*-*228* and *hco*-*mir*-*235* were designed by Exiqon (http://www.exiqon.com/) and tested together at a final concentration of 2 μM, and compared to wells containing miRCURY LNA Power Inhibitor Negative control A at the same final concentration. All experiments were carried out at least three times and statistical analysis performed using two-sample t-test. Δ7-dafachronic acid (Δ7-DA) in ethanol, was added at final concentrations of 1, 2.5, 5 and 10 μM. Control wells contained the same volume of ethanol. Larvae were examined microscopically every 24 h and number of larvae developing from L3 to L4 were counted from triplicate experiments, with total sample size between 246–367 larvae.

### Microscopy

*H*. *contortus* larvae were pipetted onto 2% agarose pads on a microscope slide. Images were captured using an Axioskop 2 Plus microscope (Zeiss), ORCA-ER digital camera (Hamamatsu) and Openlab (Improvision) software at x10 or x40 magnification. Larval development in 96 well plates was assessed using an Olympus CK2 inverted microscope at x20 magnification.

### Bioinformatic prediction of miRNA target genes and pathway analysis

Putative targets of *C*. *elegans mir*-*228* or *mir*-*235* were predicted using algorithms PITA^38^ (http://genie.weizmann.ac.il/pubs/mir07/mir07_dyn_data.html; using settings seed = 7, no G:U allowed, single mismatch allowed) and TargetScan Worm 6.0^39^. For each miRNA, only target sites predicted using both methods were retained and refined by retaining only those ranked in the top 20 of the TargetScan set and with PITA ΔΔG ≤ −7. Genes with multiple predicted target sites, not already included in these refined sets, were added if sites had PITA ΔΔG values ≤ −7. Gene functions were identified from WormBase (www.wormbase.org).

For *H*. *contortus* target gene prediction, two custom databases were generated exactly as described^65^ and when combined, contained either 3′UTR or downstream sequences for approximately 95% of *H*. *contortus* genes. The combined databases were interrogated for putative *hco*-*mir*-*228* or *hco*-*mir*-*235* binding sites using three target prediction programs: miRanda^43^, PITA^38^ and RNAhybrid^44^ that allow input of query 3′UTR sequence. Predictions were performed using the Unbuntu (14.04 LTS) operating system. Results were filtered using the following criteria: miRanda: Score >145 and energy <−10; PITA: seed sequences = 8 and ΔΔG < −10; RNAhybrid: P-value < 0.1 and energy <−22, as described^66^.

Predicted targets of *cel*-*miR*-*228* and *cel*-*mir*-*235* were used in pathway analysis using DAVID^67^ (https://david.ncifcrf.gov/) and converted into associated biological pathways based on gene-annotation enrichment analysis. P-values were adjusted for multiple testing to control the false discovery rate^63^ and pathways with P < 0.05 were considered significant. DAVID Gene Ontology (GO) analysis was also performed for *hco*-*mir*-*228* and *hco*-*mir*-*235* target genes predicted by each program individually and genes common to all three prediction programs. It was necessary to use the putative *C*. *elegans* homologous genes as input sequence, where available, identified using BLASTp (query coverage and identity >40% and best match by reciprocal BLASTp). P-value was set to 0.1 to include all potentially relevant biological pathways.

### Analysis of miRNA-mRNA interactions in HEK-293 cells

3′UTRs from six *C*. *elegans* predicted target genes (F33D4.6, *ets*-*4*, *zmp*-*2*, *mboa*-*2*, *dbt*-*1*, Y39E4A.3) were amplified from *C*. *elegans* genomic DNA using Pfu Ultra II Fusion HS DNA Polymerase (Agilent), cloned into pCR2.1 TOPO (Invitrogen) then subcloned into the *Not*I site downstream of firefly luciferase in pMirTarget (Origene) as described^45^. A region of *C*. *elegans mir*-*228* or *mir*-*235* (211 bp or 340 bp, respectively) was cloned into pCR2.1 TOPO and subcloned into vector pEGFP-N1 (Clontech) in forward and reverse orientations. All clones were verified by DNA sequencing (Eurofins Genomics, Germany). HEK-293 cells were transfected as described^45^ using 25 ng *cel*-*mir*-*228* or *cel*-*mir*-*235*-containing plasmid, 50 ng pMirTarget derived plasmid and 0.5 ng phRG-TK (Renilla luciferase, Promega). Reporter gene expression was determined as the ratio of firefly luciferase in cells transfected with miRNA in the forward/reverse direction using six replicates per test condition. Results show the mean of three replicate experiments.

### Generation of *C*. *elegans* crossed strains and production of synchronized larvae

*C*. *elegans* strains used are listed in Supplementary Table 14. A double mutant strain for *mir*-*235* and *mir*-*228* (CLB061) was generated by crossing strains MT17997(*mir*-*235*(*n4504*)*I*) and MT14446 (*mir*-*228*(*n4382*)*IV*). Seventy F1 heterozygous progeny were picked to single plates and DNA extracted and PCR amplified from F2 worms to identify those carrying homozygous deletions at both loci.

Strain CLB061 was crossed with CB1370(*daf*-*2* (*e1370*)*III*) to generate strain CLB065, which was maintained at 15 °C and homozygous deletion at each locus confirmed by PCR. Effects of *mir*-*228* and *mir*-*235* loss on *daf*-*2*(*e1370*) development were examined by placing eight L4 on NGM agar plates seeded with OP50 *E*. *coli* and allowing to egg lay overnight at 25 °C or 22.5 °C. Development of progeny to dauer or reproducing adults was examined daily. To score number of progeny in the F1 generation, eight L4 were placed on NGM/OP50 plates and progeny counted 48 hours after the start of egg laying. Triplicate plates were scored, with total sample size between 140–206.

*C*. *elegans ets*-*4*::*gfp* reporter strain RAF1713(*rrrSi400* [*Pets*-*4*::*ets*-*4*::*gfp*::*ets*-*4 3*′*UTR*] *II*.; *unc*-*119*(*ed3*) *III*)^38^ was crossed with MT17997 (*mir*-*235*(*n4504*)*I*) and F2 progeny screened by PCR to identify homozygous *mir*-*235* mutants. Synchronized L4 of RAF1713 or RAF1713 crossed into the *mir*-*235* mutant background (strain CLB072) were allowed to egg lay for 24 h on *rege*-*1* RNAi plates^40^ and GPF expression in adult F1 progeny compared. GFP expression in posterior gut nuclei was scored blind from three individual experiments, with total sample size between 120–135.

For qPCR, sixty adult *C*. *elegans* N2 hermaphrodites were allowed to egg lay overnight on NGM/OP50 plates. After 48 h, synchronized L4 were washed from plates in M9 buffer, collected by centrifugation at 6000 rpm and frozen at −80 °C. Total RNA was extracted and used in qPCR as described above.

### Quantifying dauer formation in *C*. *elegans*

Dauer arrest was assessed in *C*. *elegans* mutant strains by allowing 20 gravid adult worms to lay eggs on NGM agar plates containing 2% (v/v) crude dauer pheromone extract, prepared as described^68^. After 4–6 hours, adults were removed and plates incubated at 27 °C for 72 hours. Temperature and pheromone concentration were optimised to induce high levels of dauer formation in N2 wild-type strain, as described^68^, enabling detection of any reduction in dauer formation in mutant strains. A temperature of 27 °C was applied to obtain high numbers of dauers; this was previously used to identify novel genes involved in dauer arrest and did not lead to non-specific effects^69,70^. The total number of worms on each plate was counted before plates were flooded with 1% SDS and incubated at 20 °C for 20 minutes. Worms that survived SDS-exposure, indicated by motility and possessing a dauer-like appearance, were scored as dauers. The mutant strains tested were: MT14446 (*mir*-*228*(*n4382*)*IV*); MT17997 (*mir*-*235*(*n4504*)*I*); CBL061 (*mir*-*235*(*n4504*)*I*; *mir*-*228*(*n4382*)*IV*); MT16471 (*mir*-*60*(*n4947*)*II*). Statistical significance was measured using one-way ANOVA with corrected P-value < 0.05. Triplicate plates were scored with a total sample size between 136–338 larvae.

## Supplementary information

Supplementary Information

Supplementary Figures 1 and 2

Table S1

Table S2

Table S3

Table S4

Table S5

Table S6

Table S7

Table S8

Table S9

Table S10

Table S11

Table S12

Table S13

Table S14

## Data Availability

All microarray files are available from the NCBI GEO database (Accession Number GSE101501). Gene sequence data is available from GenBank (Accession Number MN017114).
