# Supplementary material for: Profiling microRNAs through development of the parasitic nematode Haemonchus identifies nematode-specific miRNAs that suppress larval development
Source: Sci Rep. 2019 Nov 26;9:17594. doi: 10.1038/s41598-019-54154-6 (PMC6879476; doi:10.1038/s41598-019-54154-6)
Supplement: Supplementary file 1 — Supplementary Information [file 41598_2019_54154_MOESM1_ESM.docx]

**Supplementary Information**

**Manuscript title**

**Profiling microRNAs through development of the parasitic nematode *Haemonchus* identifies nematode-specific miRNAs that suppress larval development**

**Author List**

Neil D Marks

Alan D Winter

Henry Y Gu

Kirsty Maitland

Victoria Gillan

Martin Ambroz

Axel Martinelli

Roz Laing

Rachel MacLellan

Jessica Towne

Brett Roberts

Eve Hanks

Eileen Devaney

Collette Britton

**Supplementary Figure legends**

Supplementary Figure S1. Conservation of *H. contortus* miRNAs across different phyla. miRNAs were categorised into those differentially expressed between stages and/or gut tissue (right pie chart) and those with invariant expression and/or low abundance (< 500 fluorescent units; left pie chart), based on microarray data. Colour coding indicates the conservation status of miRNAs in each category.

Supplementary Figure S2. Comparison of *hco-mir-228* and *hco-mir-235* expression in L1 and L3 stages. qPCR was carried out in triplicate for each sample and the mean of duplicate samples is shown, relative to L3 stage (shown as 1). Values for each duplicate sample were similar, as shown on right hand side.

**Supplementary Table legends**

Supplementary Table S1. *H. contortus* miRNA microarray data

Supplementary Table S2. Conservation of differentially expressed *H. contortus* miRNAs in other organisms (from miRBase release 21)

Supplementary Table S3. Pairwise comparisons of miRNA levels between sequential developmental stages, or adult female extract and adult female gut tissue.

Supplementary Table S4. Predicted target genes of *C. elegans* *mir-228* using PITA or TargetScan and those common to both target prediction programs

Supplementary Table S5. Predicted target genes of *C. elegans* *mir-235* using PITA or TargetScan and those identified by both prediction programs.

Supplementary Table S6. GO enrichment terms for TargetScan and PITA predicted target genes for *C. elegans mir-228*. Statistically significant GO terms identified by DAVID (Bejamini score < 0.05).

Supplementary Table S7. GO enrichment terms for TargetScan-predicted genes of *C. elegans mir-235*.

Supplementary Table S8. Predicted target genes of *H. contortus* *mir-228* using MiRanda, PITA or RNAhybrid and those common to all three programs. *H. contortus* gene identifiers shown with corresponding WormBase (WB) Gene number for homologous *C. elegans* gene. NA indicates no identifiable *C. elegans* homolog

Supplementary Table S9. Predicted target genes of *H. contortus* *mir-235* using MiRanda, PITA or RNAhybrid and those common to all three programs. *H. contortus* gene identifiers shown with corresponding WormBase (WB) Gene number for homologous *C. elegans* gene. NA indicates no identifiable *C. elegans* homolog

Supplementary Table S10. PITA predicted binding sites for *hco-mir-228* and *hco-mir-235* in *H. contortus* putative homologs of high confidence target genes for *cel-mir-228* and *cel-mir-235*. Position refers to nucleotide downstream of predicted stop codon; genes included if PITA ΔΔG value ≤ -7

Supplementary Table S11. GO enrichment terms for predicted target genes of *H. contortus mir-228* from MiRanda, PITA or RNAhybrid or those common to all three prediction programs

Supplementary Table S12. GO enrichment terms for predicted target genes of *H. contortus mir-235* from MiRanda, PITA or RNAhybrid or those common to all three prediction programs

Supplementary Table S13. Oligonucleotide primer sequences (* indicates phosphorothioate nucleotide bonds)

Supplementary Table S14. *C. elegans* strains used in this study
