# Supplementary figures and images for: Profiling microRNAs through development of the parasitic nematode Haemonchus identifies nematode-specific miRNAs that suppress larval development
Source: Sci Rep. 2019 Nov 26;9:17594. doi: 10.1038/s41598-019-54154-6 (PMC6879476; doi:10.1038/s41598-019-54154-6)

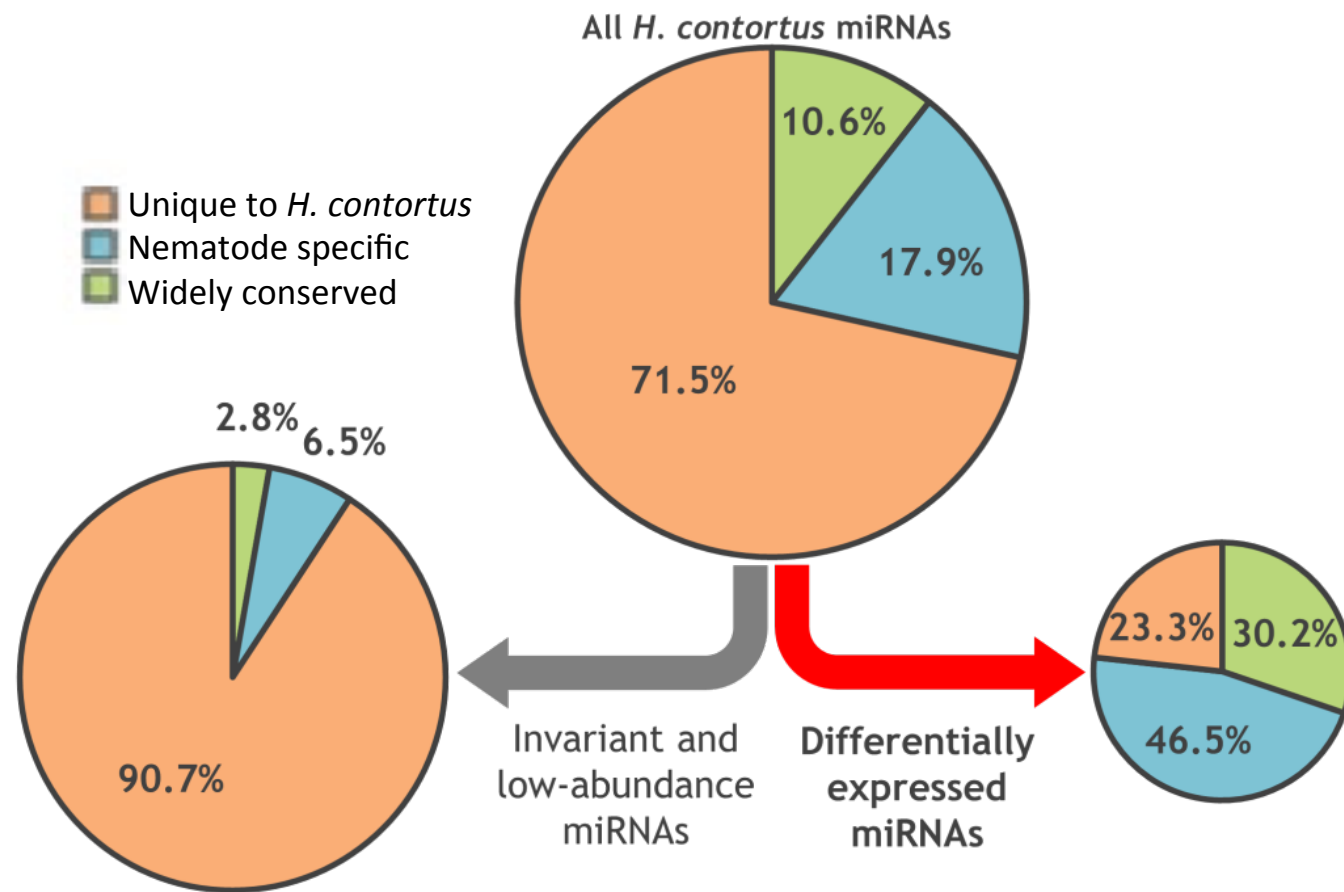

Figure S1

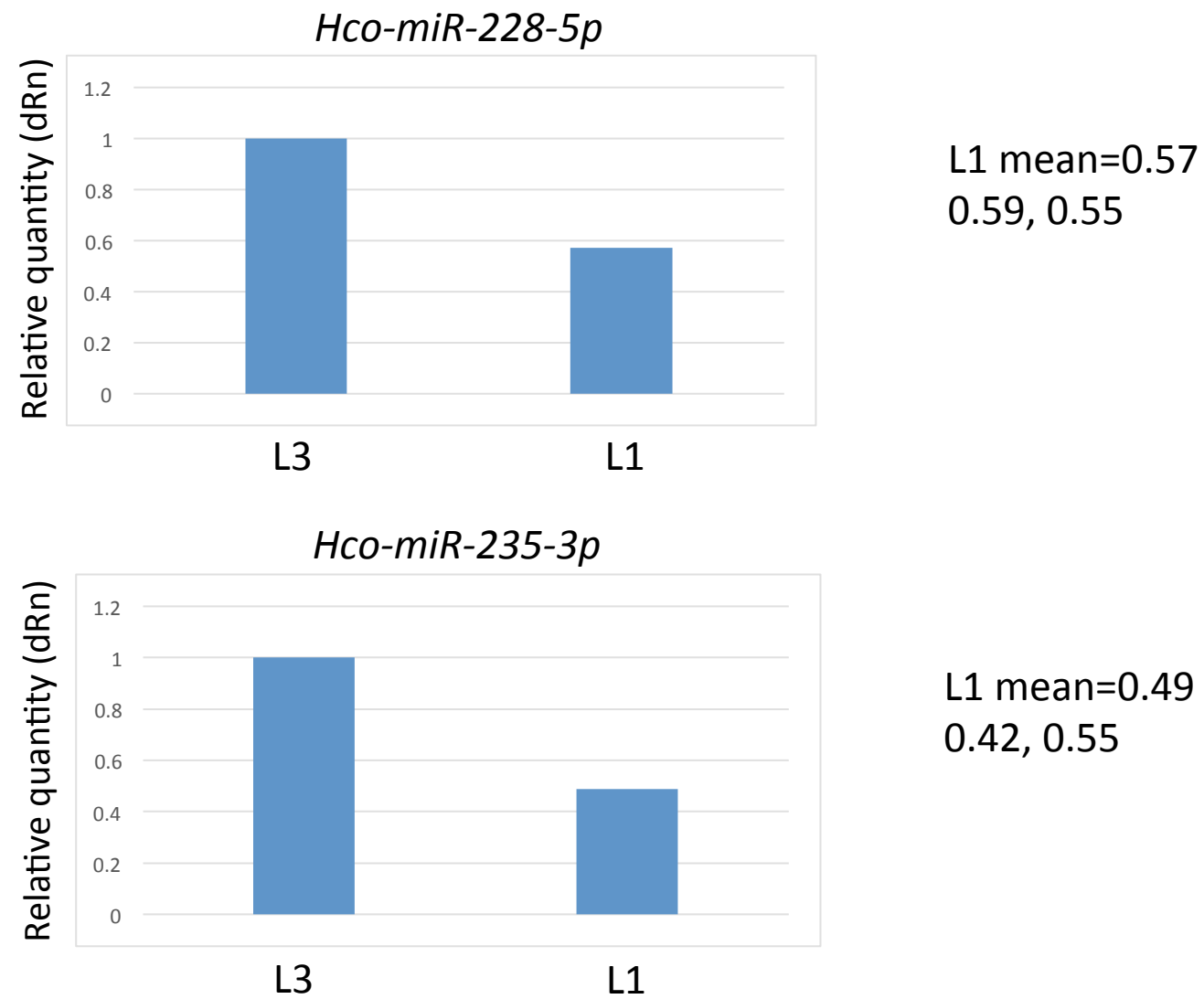

Figure S2

Supplement: Supplementary file 2 — Supplementary Figures 1 and 2 [file 41598_2019_54154_MOESM2_ESM.pdf]
