# Supplementary material for: Profiling microRNAs through development of the parasitic nematode Haemonchus identifies nematode-specific miRNAs that suppress larval development
Source: Sci Rep. 2019 Nov 26;9:17594. doi: 10.1038/s41598-019-54154-6 (PMC6879476; doi:10.1038/s41598-019-54154-6)
Supplement: Supplementary file 15 — Table S13 [file 41598_2019_54154_MOESM15_ESM.docx]

Table S13. Oligonucleotide primer sequences (* indicates phosphorothioate nucleotide bonds)

| Sequence | Description |
| --- | --- |
| TGACTAGAGACACATTCAGC | Forward primer for *hco-miR-45-3p* |
| TATTATGCACATTTTCTGGTTC | Forward primer for *hco-miR-60-3p* |
| TGAAAGACATGGGTAGTGAGAC | Forward primer for *hco-miR-71-5p* |
| GTGAGCAAAGTCTCAGGTGTGG | Forward primer for *hco-miR-87-3p* |
| AAATGGCACTGCATGAATTCACGG | Forward primer for *hco-miR-228-5p* |
| TATTGCACTCGCCCCGGCCTGA | Forward primer for *hco-miR-235-3p* |
| TACCCGTAATGTACATAGCTTGAG | Forward primer for *hco-miR-5899-3p*  Used as a normaliser in miRNA qRT-PCR |
| CGCAGCTGACGGTGTTCCAG | Forward primer for *cel-mir-235* mutant cross screening by PCR |
| CACATGATTTATCGGTATCCG | Reverse primer 1 for *cel-mir-235* mutant cross screening by PCR |
| GGAATGCACATTCTTCTGAGTTTTCG | Reverse primer 2 for *cel-mir-235* mutant cross screening by PCR |
| CCCTTACTTTTCAACGACCTCG | Forward primer 1 for *cel-mir-228* mutant cross screening by PCR |
| GCAAGCTCCGCCCATTTCTCTC | Reverse primer 1 for *cel-mir-228* mutant cross screening by PCR |
| GCCCAAATGGCCATGGGAAAG | Reverse primer 2 for *cel-mir-228* mutant cross screening by PCR |
| GGTCAACACACGCCCACCAAAAATAG | Forward primer for *C. elegans* T04A8.7 |
| TGTACACATTGGCATTCTGATCCC | Reverse primer for *C. elegans* T04A8.7 |
| TCGCCACATCTACAGTCCAATGATG | Forward primer for *Cel-mboa-2* |
| CATGTCCGTACATGAGAATTGCAAG | Reverse primer for *Cel-mboa-2* |
| GGGAGGCGACACGTTGCATGCCCAAC | Forward primer for *Cel-ets-4* |
| GCGATGGCAAAACAGTTCCCTGTTG | Reverse primer for *Cel-ets-4* |
| GATGACGAGTCGTTCAGCTTCAAGAC | Forward primer for *C. elegans* F33D4.6 |
| CGTCTCTGGTTACCGGACGATACATG | Reverse primer for *C. elegans* F33D4.6 |
| TCACGATCATGAGACCATTCA | Forward primer for *Cel-act-3* qRT-PCR normalizer |
| GCAAATTGTAGTGGGGTCTTCTTATG | Reverse primer for *Cel-act-3* qRT-PCR normalizer |
| C*C*G*G*G*G*C*G*A*G*T*G*C*A*A*T | *Hco-mir-235* miRCURY LNA Power inhibitor |
| T*T*C*A*T*G*C*A*G*T*G*C*C*A*T*T | *Hco-mir-228* miRCURY LNA Power inhibitor |
| A*C*G*T*C*T*A*T*A*C*G*C*C*C*A | Negative control miRCURY LNA Power inhibitor |

| Sequence | Description |
| --- | --- |
| CACCTCATCATTACTCAAACCAATATATTG | Forward primer for *cel-daf-2* mutant cross screening by PCR, wild-type |
| CCAATATAATTCAACACCTCATCATTACTCAAACCA  ATATATCA | Forward primer for *cel-daf-2* mutant cross screening by PCR, mutant |
| GCAAGCGTATGATGCCTGTTCGATG | Reverse primer for *cel-daf-2* mutant cross screening by PCR, mutant and wild-type |
| CGTCGACTCCCAAAAACTGTTTCGCAAC | Forward primer for *cel-mir-228* in pEGFP-N1 (SalI) |
| GTCGACCAATGTCTCCTGCTCTTTTTCCCAC | Reverse primer for *cel-mir-228* in pEGFP*-*N1 (SalI) |
| GGTAACCAATCGAAACTCGTTTTC | Forward primer for *cel-mir-235* in pEGFP-N1 (KpnI) |
| GGTACCACAGAGTATATAAAGAAACCTGC | Reverse primer for *cel-mir-235* in pEGFP-N1 (KpnI) |
| GCGGCCGCTTAATTAATACCTTTATATTAATTTTTA  TTTTG | Forward primer for F33D4.6 in pMirTarget (NotI) |
| GCGGCCGCGAATAACCATTTATTAACCATG | Reverse primer for F33D4.6 in pMIrTarget (NotI) |
| GCGGCCGCTCATCTGGCAGAAAGACAACGAC | Forward primer for *cel-ets-4* in pMirTarget (Not) |
| GCGGCCGCAATATTTATCTGGTTCATTAAT | Reverse primer for *cel-ets-4* in pMirTarget (NotI) |
| GCGGCCGCACCCTTCATCACCTCCATCCATAG | Forward primer for *cel-zmp-2* in pMirTarget (NotI) |
| GCGGCCGCTTTTATTGCCATCATTGATTTGAAC | Reverse primer for *cel-zmp-2* in pMirTarget (NotI) |
| GCGGCCGCTTTATCTCAATTTGTTTTTTTTTAATTAG | Forward primer for *cel-mboa-2* in pMirTarget (NotI) |
| GCGGCCGCGAAAAAATAAATTTTATTCACACAG | Reverse primer *cel-mboa-2* in pMirTarget (NotI) |
| GCGGCCGCAGTATCATGTTTCATCTGGTTAAT | Forward primer for *cel-dbt-1* in pMirTarget (NotI) |
| GCGGCCGCTGGAACTCGATACAAAGAAGCC | Reverse primer for cel-dbt-1 in pMirTarget (NotI) |
| GCGGCCGCATTCATTTTTTGTGATGCTTTTT | Forward primer for Y39E4A.3 in pMirTarget (NotI) |
| GCGGCCGCTTGGTTGTTCAGTGGCAATAAATTG | Reverse primer for Y39E4A.3 in pMirTarget (NotI) |
| CAAGAAGCTGCGCGGTGGTG | Forward pMir-Target for PCR screening |
| GATGGCAGGTTGGGCGTCGC | Reverse pMir-Target for PCR screening |
| GTCGTAACAACTCCGCCC | Forward pEGFP-N1 for PCR screening |
| GTCCAGCTCGACCAGGATG | Reverse pEGFP-N1 for PCR screening |
