# Supplementary material for: Profiling microRNAs through development of the parasitic nematode Haemonchus identifies nematode-specific miRNAs that suppress larval development
Source: Sci Rep. 2019 Nov 26;9:17594. doi: 10.1038/s41598-019-54154-6 (PMC6879476; doi:10.1038/s41598-019-54154-6)
Supplement: Supplementary file 16 — Table S14 [file 41598_2019_54154_MOESM16_ESM.docx]

| Strain | CGC/Britton lab number |
| --- | --- |
| N2 bristol | **N2** |
| *mir-228(n4382)IV* | **MT14446** |
| *mir-235(n4504)I* | **MT17997** |
| *mir-235(n4504)I; mir-228(n4382)IV* | CLB061 |
| *daf-2 (e1370)III* | **CB1370** |
| *mir-235(n4504)I; daf-2 (e1370)III; mir-228(n4382)IV* | CLB065 |
| *rrrSi400 [Pets-4::ets-4::gfp::ets-4 3'UTR] II; unc-119(ed3) III* | RAF1713 |
| *mir-235(n4504)I; rrrSi400 [Pets-4::ets-4::gfp::ets-4 3'UTR] II; unc-119(ed3) III* | CLB072 |
| *mir-60(n4947)II* | **MT16471** |
| *daf-16(mu86) I; daf-2(e1370) III; muIs84* | **CF1588** |

Table S14. *C. elegans* strains used in this study
